# Incidence, Risk Factors and Mortality Associated with Major Bleeding Events in Hospitalized COVID-19 Patients

**DOI:** 10.3390/life13081699

**Published:** 2023-08-07

**Authors:** Marko Lucijanic, Ida Tjesic-Drinkovic, Nevenka Piskac Zivkovic, Frane Pastrovic, Zrinka Rob, Mersiha Bacevac, Martina Sedinic Lacko, Eleonora Dzambas, Barbara Medic, Ivan Vukoja, Iva Busic, Ivica Grgurevic, Ivica Luksic, Bruno Barsic

**Affiliations:** 1 Hematology Department, University Hospital Dubrava, 10000 Zagreb, Croatia; sedinicm@gmail.com; 2 School of Medicine, University of Zagreb, 10000 Zagreb, Croatia; 3 Department of Gastroenterology, Hepatology and Clinical Nutrition, University Hospital Dubrava, 10000 Zagreb, Croatia; 4 Pulmonology Department, University Hospital Dubrava, 10000 Zagreb, Croatia; 5 Department of Emergency and Intensive Care Medicine, University Hospital Dubrava, 10000 Zagreb, Croatia; 6 Gastroenterology and Nephrology Department, General County Hospital Pozega, 34000 Pozega, Croatia; 7 Faculty of Medicine Osijek, Josip Juraj Strossmayer University of Osijek, 31000 Osijek, Croatia; 8 Department of Maxillofacial Surgery, University Hospital Dubrava, 10000 Zagreb, Croatia

**Keywords:** coagulopathy, thrombo-inflammation, SARS-CoV-2, IL-6, low molecular weight heparin

## Abstract

Thromboprophylaxis is a mainstay of treatment of hospitalized COVID-19 patients, due to the high occurrence of thrombotic events. This increases the risk of bleeding. However, data on bleeding events and associated risk factors are scarce. Thus, we aimed to investigate the incidence, predictors and clinical outcomes associated with major bleeding in hospitalized COVID-19 patients. We retrospectively evaluated a cohort of 4014 consecutively hospitalized COVID-19 patients treated in a tertiary-level institution in the period 3/2020–3/2021. Bleeding of any kind was documented in 322 (8%) and major bleeding in 129 (3.2%) patients. A total of 129 (40.1%) bleeding events were present at the time of hospital admission, and 193 (59.9%) occurred during hospitalization. In the multivariate logistic regression analysis, intensive-care-unit treatment (adjusted odds ratio (aOR) 6.55; *p* < 0.001), atrial fibrillation (aOR 2.55; *p* = 0.029), higher white-blood-cell count (WBC) (aOR 1.03; *p* = 0.021), lower hemoglobin (aOR 0.97; *p* = 0.002) and history of bleeding (aOR 17.39; *p* < 0.001) were recognized as mutually independent predictors of major bleeding. Major bleeding was significantly associated with increased in-hospital mortality compared to non-major-bleeding patients (59.7% vs. 34.8%, *p* < 0.001), especially if occurring during hospitalization. Median time from major bleeding to death was 5 days. Bleeding events are frequent in hospitalized COVID-19 patients, with a significant proportion of patients presenting at the time of hospital admission, and others almost universally exposed to anticoagulant and corticosteroid therapies. Major bleeding is associated with high mortality, especially if occurring during hospitalization. The recognition of patients at risk and implementation of timely interventions are of high clinical importance.

## 1. Introduction

Coronavirus disease 2019 (COVID-19) affects multiple organ systems, and may result in a high proportion of patients who require hospitalization due to development of respiratory insufficiency or other complications [1]. Although respiratory symptoms dominate the clinical presentation, many patients concomitantly develop gastrointestinal, cardiovascular and neurological symptoms, features of coagulopathy, inflammation and liver damage [2]. All these changes are more frequent with higher severity of COVID-19 symptoms, and are driven by overproduction of inflammatory molecules such as interleukin (IL) 6, IL 10, tumor necrosis factor alfa (TNF alfa), interferon gamma, etc. [3]. Strong inflammatory atmosphere associated with disease is especially pronounced in older patients with previously present comorbidities [4,5]. Continuous and uncontrolled inflammatory response to the infection, termed cytokine storm, may lead to acute respiratory distress syndrome (ARDS) and multiorgan failure [6]. ARDS is characterized by diffuse inflammatory damage to alveoli and their capillary network [7], and is the leading cause of death of COVID-19 patients presenting with severe and critical symptoms. Plasma and immune-cell profiling of patients suffering from post-acute sequelae of COVID-19 demonstrated ongoing neutrophil activity, B-cell memory alterations, and autoreactivity, even more than a year after acute infection [8].

With the introduction of vaccination, the proportion of patients with severe or critical disease presentation significantly reduced, as well as the outcomes of hospitalized patients who developed COVID-19, despite being vaccinated, significantly improving [9]. Nevertheless, issues like vaccine hesitancy, occurrence of novel severe acute respiratory syndrome coronavirus 2 (SARS-CoV-2) viral strains and waning immune response limit the benefits of vaccination [10,11,12]. This problem may be more pronounced in specific ethnic (American Indian or Alaska Native) and other socially vulnerable groups (persons with a higher community-level social vulnerability index) [12,13]. Due to the inability of vaccines to completely prevent development of COVID-19, breakthrough infections occur. Thus, COVID-19 still remains an important health-care issue.

Thrombo-inflammation is a unique feature of COVID-19 [14,15], and high frequency of arterial and venous thromboses are observed in multiple cohorts of patients [16,17]. Prothrombotic abnormalities of peripheral blood (like decreased protein C activity, decreased protein S, ADAMTS13 antigen and higher von Willebrand factor levels) are common, but do not seem to directly correlate with thrombin generation potential [15]. Thrombotic events seem to be associated with features of more severe disease, and are often subclinical in presentation in the case of venous thromboembolism (VTE) [16,18,19]. This is mostly due to an overlapping clinical presentation of both severe COVID-19 and pulmonary embolism, and may be impossible to evaluate properly in clinically unstable patients who are unable to undergo diagnostic procedures [16]. In addition to medical consequences for the affected patient per se, VTE may lead to prolonged hospital stay and impair the hospital bed network, reflecting the high number of patients who require hospital medical care [20]. With the introduction of screening methods, much higher proportions of patients with VTE are identified [18]. Nevertheless, the overwhelming character of the COVID-19 pandemic disables the utilization of screening procedures on a large scale. Several prognostic scores for VTE developed prior to the pandemic found their place in clinical decision making in the COVID-19 context. Neither of them seems to demonstrate acceptable discriminatory properties [21].

Low-molecular-weight heparin (LMWH) thromboprophylaxis was shown to significantly improve outcomes of hospitalized patients with COVID-19 [22]; however, optimal anticoagulation doses (prophylactic, intermediate- or full-therapeutic) and anticoagulant drugs of choice in patients who otherwise do not have an indication for anticoagulation still remain controversial. A more intensive anticoagulation strategy inevitably leads to higher occurrence of bleeding complications, but seems to be needed to prevent respiratory deterioration, especially among non-critical COVID-19 patients [23].

Bleeding complications in COVID-19 patients are increasingly being recognized, and are potentiated with both disease-related and iatrogenic processes [24,25]. Similar to thrombotic events, bleeding seems to be more common among critical COVID-19 patients, and balancing thrombosis and hemostasis may present a significant challenge [26]. A variety of other factors besides anticoagulation use and severity of disease may affect major bleeding, such as prior comorbidities and history of bleeding [27]. Bleeding is considered to present a less-prominent clinical problem in comparison to thrombotic events. Gastrointestinal bleeding and intracranial hemorrhage may be of special concern, due to clinical consequences and potentially modifiable risks that may cause them (hypoxia, ulcerogenic and bleeding contribution of different drugs, drug-to-drug interactions, etc.) [28].

Uncertainties still exist about incidence and risk factors for bleeding complications, especially major bleeding complications in hospitalized COVID-19 patients. Thus, we aimed to investigate the occurrence of bleeding events, associated clinical characteristics, and outcomes in a real-life cohort of hospitalized COVID-19 patients from our institution.

## 2. Materials and Methods

A total of 4014 consecutive COVID-19 patients hospitalized in our tertiary level institution in the period from March 2020 to March 2021 were retrospectively evaluated for their baseline clinical profile and occurrence of bleeding complications. During the study period, our institution was completely repurposed to serve as referral center for treatment of most severe COVID-19 patients and those who had other acute medical conditions and were concomitantly SARS-CoV-2 positive. All patients were white adults. All patients had a positive polymerase chain reaction (PCR) or antigen COVID-19 test, with compatible clinical symptoms. Patients were treated according to the contemporary guidelines, with the majority of patients (85.9%) receiving pharmacologic LMWH thromboprophylaxis with various dose intensities, in line with the individual judgement of treating physicians. Dalteparin doses of up to 5000 units once daily and enoxaparin doses of up to 40 mg once daily were considered as prophylactic, and above these as intensified LMWH dosing. The majority of patients received corticosteroids. Corticosteroid doses in the range 1–2 mg prednisone equivalent per kg body weight or higher were considered as intensified corticosteroid therapy. Clinical and laboratory characteristics of evaluated patients were obtained through analysis of electronic and written medical records, as a part of a hospital registry project. COVID-19 disease severity on admission was graded as mild, moderate, severe and critical, according to the World Health Organization [29]. The Eastern cooperative oncology group (ECOG) scale was used to determine the functional status of patients, on admission [30]. Comorbidities were evaluated as particular entities and as cumulative comorbidity burden, estimated using the Charlson comorbidity index [31].

We have evaluated the following demographic and clinical parameters: age, sex, origin of referral (home, nursing home, other hospital), day of disease on admission, ECOG status on admission, pneumonia, bilateral pneumonia, oxygen therapy, COVID severity, other infection on admission, length of hospitalization (days), intensive care unit, high-flow oxygen therapy (requiring use of high-flow nasal cannula to deliver oxygen at flows above 15 L/min), mechanical ventilation, immobilization ≥7 days, VTE, pulmonary embolism, deep venous thrombosis, arterial thrombosis, acute myocardial infarction, acute cerebrovascular insult, LMWH therapy, therapeutic LMWH therapy, corticosteroid therapy, and intensified corticosteroid therapy.

We evaluated the following comorbidities and specific therapies prior to admission: arterial hypertension, diabetes mellitus, hyperlipoproteinemia, obesity, metabolic syndrome, congestive heart failure, atrial fibrillation, coronary artery disease, peripheral artery disease, history of myocardial infarction, history of cerebrovascular insult, history of VTE, history of bleeding, chronic kidney disease, chronic hemodialysis, gastroesophageal reflux disease/peptic ulcer disease, inflammatory bowel disease, chronic liver disease, liver cirrhosis, epilepsy, mental retardation, schizophrenia, dementia, active malignant disease, metastatic malignant disease, history of malignant disease, thyroid disease, autoimmune/rheumatic disease, asthma, chronic obstructive pulmonary disease, transplanted organ, trauma/surgery one month prior to or during hospitalization, known thrombophilia, prior anticoagulant therapy, aspirin, steroids prior to admission, antipsychotics, antidepressants, active chemotherapy, and statin and hormonal therapy.

We have evaluated, following laboratory parameters: IL-6 (pg/mL), procalcitonin (ng/mL), white blood cell count (WBC) (x109/L), hemoglobin (g/L), mean corpuscular volume (MCV) (fL), mean corpuscular hemoglobin concentration (MCHC) (g/L), red blood cell distribution width (RDW) (%), platelets (x109/L), C-reactive protein (CRP) (mg/L), ferritin (µg/L), D-dimers (mg/L fibrin equivalent units), estimated glomerular filtration rate (eGFR) (mL/min/1.73 m^2^), lactate dehydrogenase (LDH) (U/L), aspartate aminotransferase (AST) (U/L), alanine aminotransferase (ALT) (U/L), gamma-glutamyl transferase (GGT) (U/L), alkaline phosphatase (ALP) (U/L), total bilirubin (µmol/L), albumin (g/L), and prothrombin time (PT) (%).

Clinically relevant bleeding events, as well as major bleeding events, had to be documented during hospitalization or at the time of hospital admission. Major bleeding was defined according to the International Society on Thrombosis and Haemostasis (ISTH) criteria [32]. The following bleeding localizations were considered: gastrointestinal tract, respiratory tract, urinary tract, intracranial, intramuscular, cutaneous, vaginal, iatrogenic, and postsurgical and internal bleeding. Due to low frequency, iatrogenic, postsurgical and internal bleeding events were evaluated as “other”.

The study was approved by the University Hospital Dubrava Review Board (nm. 2021/2503-04).

### Statistical Methods

Normality of distribution of numerical variables was tested using the Kolmogorov–Smirnov test. Due to non-normal distribution, numerical variables were presented as median and interquartile range (IQR), and were compared using the Mann–Whitney U test and the Kruskal–Wallis ANOVA. Categorical variables were presented as frequencies and percentages, and were compared using the Χ2 test. Platelet counts on admission and at the time of bleeding were compared using the Wilcoxon test for paired samples. Associations of clinical parameters with occurrence of major bleeding were assessed using the logistic regression analysis. For the assessment of independent associations with major bleeding occurrence, a model-building process was performed, using the backward approach with *p* < 0.05 and *p* > 0.1 criteria for variable inclusion and removal, respectively. *p* values < 0.05 were considered statistically significant. All analyses were performed using the MedCalc statistical software, version 20.006 (MedCalc Software Ltd., Ostend, Belgium).

## 3. Results

### 3.1. Overview of Patients’ Characteristics and Bleeding Events

We analyzed a total of 4014 hospitalized COVID-19 patients. The median age was 74 years IQR (64–82), median Charlson comorbidity index was 4 IQR (3–6), and 2256 (56.2%) patients were males. Median duration of COVID-19 at the time of hospital admission was 5 days IQR (1–9). Severe or critical disease was present in 3359 (83.7%) patients at the time of admission. During hospitalization, 913 (22.7%) required intensive-care-unit treatment, 771 (19.2%) required high-flow oxygen therapy, 675 (16.8%) required mechanical ventilation, and 1428 (35.6%) patients died.

Bleeding of any kind was documented in 322 (8%) and major bleeding in 129 (3.2%) patients. Bleeding of any kind was present in 9.1% patients with mild, 6.3% with moderate, 7.3% with severe, and 11% with critical COVID-19, on admission (*p* = 0.013). Severe and critical compared to non-severe COVID-19 patients had similar rates of bleeding-of-any-kind events (8% vs. 8.2%, *p* = 0.819). Major bleeding was present in 3.6% patients with mild, 1.9% with moderate, 2.8% with severe, and 5.4% with critical COVID-19, on admission (*p* = 0.008). Severe and critical compared to non-severe COVID-19 patients had similar rates of major bleeding events (3.2% vs. 3.1%, *p* = 0.799). ICU-treated patients had higher bleeding of any kind (13.3% vs. 6.5%, *p* < 0.001) and major bleeding rates (7.4% vs. 2%, *p* < 0.001) compared to non-ICU-treated ones.

**Figure 1:**
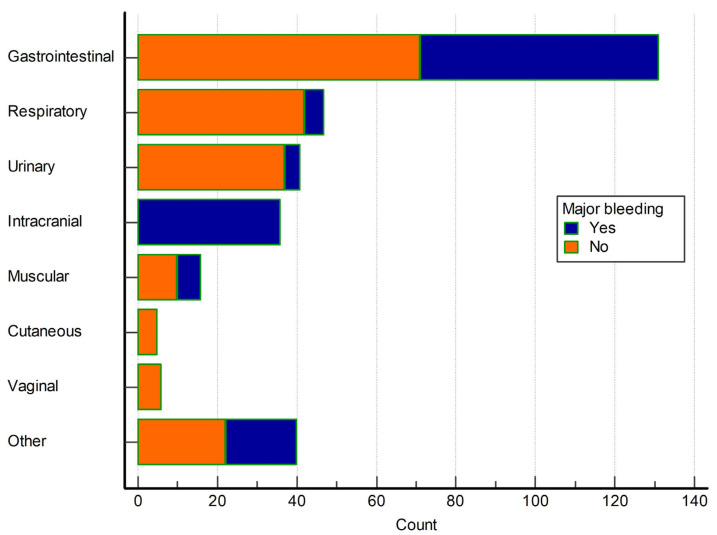
Frequencies of non-major and major bleeding events, stratified according to the bleeding localizations.

**Figure 2:**
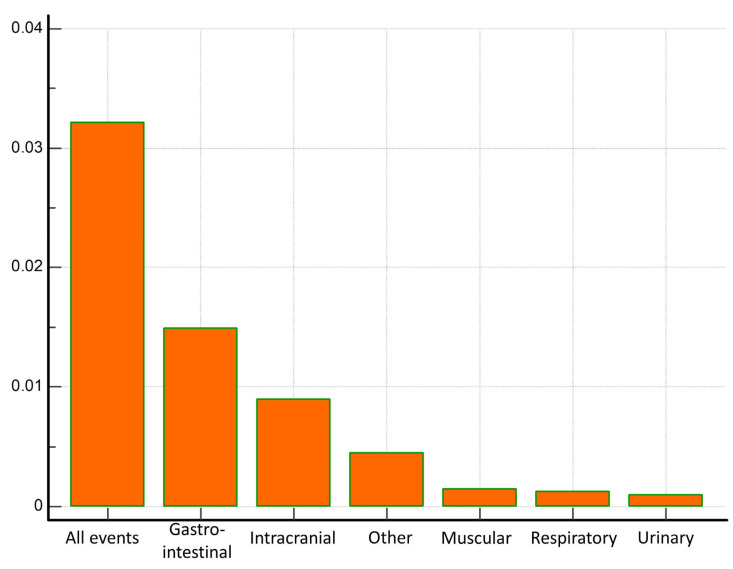
Incidence rates of major bleeding events for all events, and stratified according to the bleeding localizations.

The most common bleeding localizations were the gastrointestinal tract, in 131 (40.7% of all events), the respiratory tract in 47 (14.6% of all events), the urinary tract in 41 (12.7% of all events), intracranial in 36 (11.2% of all events), intramuscular in 16 (5% of all events), cutaneous in 6 (1.9% of all events), vaginal in 5 (1.6% of all events) and other (iatrogenic, postsurgical and internal bleeding events) in 40 (12.4% of all events). Major bleeding events were present in 60 (45.8%) patients with gastrointestinal tract bleeding, 5 (10.6%) with respiratory tract, 4 (9.8%) with urinary tract, 36 (100%) with intracranial bleeding, 6 (37.5%) with intramuscular bleeding, none with cutaneous and vaginal bleeding, and 18 (45%) with other types of bleeding. Proportions of major bleeding among bleeding events stratified according to the bleeding localizations are depicted in Figure 1. Incidence rates of major bleeding events in general, and stratified according to the bleeding localizations, are depicted in Figure 2.

A total of 129 (40.1%) bleeding events were present on admission, and 193 (59.9%) occurred during hospitalization. Median time of post-admission bleeding occurrence was 7 days IQR (3–14), without significant differences associated with different bleeding localizations or with bleeding severity. Regarding bleeding localizations, there were no significant differences in the proportions of gastrointestinal bleeding events (50.4% vs. 49.6%) or vaginal bleeding events (66.7% vs. 33.3%) occurring at the time of and after admission, whereas there were a significantly higher proportion of intracranial bleeding events (69.4% vs. 30.6%) and significantly lower proportions of respiratory tract (21.3% vs. 78.7%), urinary tract (34.1% vs. 65.9%), muscular (18.8% vs. 81.2%), cutaneous (40% vs. 60%) and other bleeding events (12.5% vs. 87.5%) occurring at the time of hospital admission, in comparison to later on during hospitalization (*p* < 0.05 for aforementioned analyses). Major bleeding events were similarly distributed at the time of and after admission (46.5% vs. 53.5%).

Platelet counts at the time of hospital admission had no significant association with occurrence of bleeding or major bleeding, and similar levels of platelets were present at the time of an event occurrence and at the time of hospital admission (median 214 vs. 213; *p* = 0.618). However, there were significant differences in platelet counts at the time of major bleeding occurrence, associated with different bleeding localizations (median 231 for gastrointestinal tract, 122 for respiratory tract, 165.5 for intramuscular, 249 for urinary tract, 157 for intracranial and 207 for other major bleeding localizations; *p* = 0.004). A significant difference in admission and time-of-event platelet counts was present only in patients experiencing major bleeding from the respiratory tract (median 154 vs. 122; *p* = 0.043).

The relationships of major bleeding with patients’ demographic characteristics, comorbidities and selected drugs are shown in Table 1, with laboratory parameters on admission in Table 2 and with COVID-19 disease severity- and hospitalization-related parameters in Table 3.

### 3.2. Factors Associated with Major Bleeding

Considering general characteristics, comorbidities and drugs in chronic therapy, major bleeding events were significantly associated (*p* < 0.05 for all analyses) with higher Charlson comorbidity index (median 5 vs. 4 points), atrial fibrillation (major bleeding 5.1% vs. 2.8%), history of bleeding (major bleeding in 36.2% vs. 2.6%), GERD/ulcer disease (major bleeding in 6% vs. 2.8%), active malignant disease (5.1% vs. 3%), metastatic malignant disease (3.6% vs. 3.2%), trauma or surgery one month prior to or during hospitalization (5.7% vs. 2.8%) and anticoagulant therapy prior to admission (4.7% vs. 2.6%). Regarding laboratory parameters on admission, major bleeding events were significantly more frequent in patients with higher procalcitonin (median 0.34 vs. 0.21), higher WBC (median 9.5 vs. 7.9), higher RDW (median 14.6 vs. 14.1), lower hemoglobin (median 114 vs. 128), lower albumin (median 29 vs. 32), lower eGFR (59.3 vs. 72) and lower PT (97% vs. 100%). Regarding COVID-19 disease severity- and hospitalization-related parameters, major bleeding events were significantly associated with referral from other hospital (major bleeding 4.3% vs. 2.1%), worse ECOG functional status on admission (median 3 vs. 3), presence of another infection on admission (4.8% vs. 2.9%), acute arterial thrombotic events (6% vs. 3%), longer duration of hospitalization (median 14 vs. 10 days), need for intensive care unit (major bleeding 7.4% vs. 2%), high-flow oxygen therapy (major bleeding 5.1% vs. 2.8%), mechanical ventilation (major bleeding 7.3% vs. 2.4%) and prolonged immobilization ≥7 days (major bleeding 4.8% vs. 2%) and therapeutic LMWH use (major bleeding 4% vs. 2.8%). There were no significant associations of major bleeding occurrence with either age, sex, IL-6 levels, CRP, platelet count on admission, presence of pneumonia, need for oxygen supplementation therapy, LMWH use, or with COVID-19 severity of symptoms on presentation.

In the multivariate logistic regression analysis performed using the backward approach and including univariately significant predictors, age, sex, intensified corticosteroid therapy and COVID-19 disease severity on admission, the parameters that remained mutually independently associated with major bleeding events were: intensive care unit treatment (adjusted odds ratio (aOR) 6.55, 95% CI (3.04–14.12); *p* < 0.001), atrial fibrillation (aOR 2.55, 95% CI (1.09–5.91); *p* = 0.029), higher WBC (aOR 1.03, 95% CI (1.01–1.069); *p* = 0.021), lower hemoglobin (aOR 0.97, 95% CI (0.96–9.99); *p* = 0.002) and history of bleeding (aOR 17.39, 95% CI (5.52–54.77); *p* < 0.001).

### 3.3. Major Bleeding and Mortality

Major bleeding occurrence was significantly associated with increased in-hospital mortality with 59.7% patients with and 34.8% patients without major bleeding dying during hospitalization (*p* < 0.001), with an especially higher risk of death if major bleeding occurred later during hospitalization (mortality 48.3% in events at the time of and 69.6% in events after hospital admission; *p* = 0.015). There were significant differences in types of major bleeding localizations associated with increased mortality (mortality 53.3% in gastrointestinal tract, 100% in respiratory tract, 66.7% in intramuscular, 25% in urinary tract, 58.3% in intracranial and 77.8% in other major bleeding localizations; *p* < 0.001). However, death did not occur immediately adjacent to bleeding events, and median time from major bleeding to death was 5 days, IQR (2–12), with no significant difference between localizations (*p* = 0.326).

## 4. Discussion

Despite the widespread use of anticoagulant therapy in hospitalized COVID-19 patients, risk factors and outcomes associated with major bleeding are not well understood. There are several important points we would like to emphasize.

Clinical focus in COVID-19 patients is placed on survival and risk of thrombotic events, with pharmacologic thromboprophylaxis, often in full therapeutic doses, being one of the mainstays of treatment. Major bleeding rates in our study were 3.2% in an overall cohort and 7.4% in ICU-treated patients, which is comparable to some of previously reported rates [33]. Anticoagulants and corticosteroid use predispose bleeding events in COVID-19 patients [34], of which about half occurred on admission and half during hospitalization (median 7th day of hospitalization) in our cohort of patients. Proportions of bleeding events on admission and during hospitalization are similar for overall and major bleeding events; however, certain localizations (respiratory, urinary tract, muscular and cutaneous localization) were more likely to be associated with occurrence during hospitalization and after ICU admission, and thus iatrogenic interventions might predispose them. On the other hand, the dose of anticoagulation provided during hospitalization could not have an effect on the occurrence of half of all bleeding events. It should be noted that rates of bleeding and major bleeding events had a U-shaped relationship with the severity of COVID-19 at the time of admission, and were higher among patients presenting with mild compared to moderate and severe COVID-19 symptoms, probably presenting an indication for hospitalization in a subset of these patients. Specific bleeding localizations were associated with a high proportion of major bleeding, like intracranial (100%), gastrointestinal (45.8%), iatrogenic, postsurgical, and internal (45%) and intramuscular (37.5%) bleeding, highlighting the importance of recognition of these types of events. Respiratory (10.6%) and urinary tract (9.8%) bleedings presented as major in a non-negligible proportion of patients, probably due to iatrogenic interventions associated with care for anticoagulated patients with respiratory insufficiency and poor performance status.

The occurrence of major bleeding is associated with higher mortality, especially if occurring during hospitalization. It is important to highlight that major bleeding was rarely the proximate cause of death, and death occurred a median 5 days after the major bleeding in the case of fatal events. Thus, bleeding events were timely and successfully resolved, but underlying risk factors, associated blood loss and further respiratory deterioration might weaken affected patients and predispose death. Major bleeding substantially contributed to worse functional status, and should be considered as an indicator of overall severity of COVID-19, with important prognostic implications regarding delayed increase in mortality.

Platelets are important mediators of hemostasis, and thrombocytopenia can be encountered in 20% of COVID-19 patients at the time of admission. Thrombocytopenia is associated with earlier presentation during disease course, higher comorbidity burden, and septic complications, as well as with the occurrence of major bleeding in COVID-19 patients [25]. Nevertheless, in the current study, platelet counts on admission did not significantly differ from those at the time of bleeding events, nor were they associated with occurrence of bleeding events. Specific major bleeding localizations like the respiratory tract were associated with a significant downward trend in platelet count from admission, and these patients had the lowest platelet count at the time of major bleeding in comparison to other localizations. The upper respiratory tract in severe and critical COVID-19 patients is exposed to non-physiologic air flow and composition, and is irritated by the installments required to provide respiratory supplementation. Lower platelet count, in addition to LMWH thromboprophylaxis and physical effects, may both contribute to high occurrence of these bleeding events.

Intensive care unit treatment, atrial fibrillation, higher WBC, lower hemoglobin, and history of bleeding were recognized as mutually independent predictors of major bleeding events. The majority of these factors result in the need for full-dose anticoagulation or additional antiplatelet therapy in hospitalized patients, potentiating the occurrence of major bleeding. A full-dose anticoagulation, although significantly associated with major bleeding events, did not demonstrate independent predicting potential in the context of the aforementioned variables. Also, an intensive care unit stay requires intensive therapeutic approaches with higher doses and a prolonged course of corticosteroid and other therapies, and results in higher functional impairment and the prolonged hospitalization of these patients. Hemoglobin reflects hematocrit, which is recognized as a surrogate for blood viscosity [35]. Red and white blood cells actively participate in the process of thrombus formation and dissolution [36], and their levels at the time of hospital admission seem to be independent predictors of a major bleeding occurrence.

It should be noted that incidence and risk factors for major bleeding might be population specific, and reflect both the demographic and comorbidity profile of patients, as well as specificities of a tertiary institution. The main limitations of our work are single-center experience, retrospective-study design and heterogenous patient population regarding exposure to LMWH, antiplatelet agents and other specific therapies. The mMain strength of our work is a large, well-described cohort of hospitalized COVID-19 patients, representative of a tertiary referral center experience with high frequency of severe and critical COVID-19 patients.

## 5. Conclusions

Overall bleeding and major bleeding events are frequent in hospitalized COVID-19 patients, due especially to the almost universal exposure to anticoagulant therapies. About half of these events occur at the time of hospital admission, whereas the other half occur during hospital stay. The occurrence of major bleeding, especially during hospitalization, is associated with high mortality, but is rarely the proximate cause of death. Recognition of patients who are at risk of major bleeding, and the implementation of timely preventive and therapeutic interventions, are of high clinical importance. Our study recognized the need for intensive care unit treatment, atrial fibrillation, history of bleeding, higher leukocytes, and lower hemoglobin, as mutually independent predictors of major bleeding events. Hospitalized COVID-19 patients with these characteristics should be given special attention regarding signs of bleeding, with the aim of the institution of appropriate and timely measures.

## Figures and Tables

**Table 1:** Patients’ demographic characteristics, comorbidities and selected drugs in chronic therapy, and their relationship with major bleeding.

	Overall (N = 4014)	OR with 95% CI for Major Bleeding
Age	74 IQR (64–82)	OR 1.01 (0.99–1.02); *p* = 0.202
Male sex	2256 (56.2%)	OR 1.12 (0.79–1.6); *p* = 0.528
Charlson comorbidity index	4 IQR (3–6)	OR 1.09 (1.03–1.16); *p* = 0.005 *
Alcohol use	218 (5.4%)	OR 1.48 (0.77–2.88); *p* = 0.240
Smoking	231 (5.8%)	OR 0.79 (0.35–1.82); *p* = 0.585
Number of drugs in chronic th.	5 (2–8)	OR 1.03 (0.98–1.07); *p* = 0.236
Arterial hypertension	2771 (69%)	OR 1.21 (0.82–1.79); *p* = 0.339
Diabetes mellitus	1201 (29.9%)	OR 0.97 (0.66–1.44); *p* = 0.907
Hyperlipoproteinemia	954 (23.8%)	OR 1.11 (0.74–1.65); *p* = 0.623
Obesity	1069 (26.6%)	OR 0.83 (0.55–1.25); *p* = 0.379
Metabolic syndrome	799 (19.9%)	OR 0.92 (0.58–1.44); *p* = 0.707
Congestive heart failure	649 (16.2%)	OR 1.13 (0.71–1.78); *p* = 0.603
Atrial fibrillation	721 (18%)	OR 1.88 (1.27–2.78); *p* = 0.002 *
Coronary artery disease	613 (15.3%)	OR 1.02 (0.63–1.65); *p* = 0.941
Peripheral artery disease	281 (7%)	OR 1.12 (0.58–2.16); *p* = 0.734
History of myocardial infarction	366 (9.1%)	OR 1.02 (0.56–1.87); *p* = 0.941
History of cerebrovascular insult	469 (11.7%)	OR 1.58 (0.98–2.53); *p* = 0.056
History of VTE	193 (4.8%)	OR 1.7 (0.88–3.29); *p* = 0.116
History of bleeding	69 (1.7%)	OR 20.98 (12.37–35.58); *p* < 0.001 *
Chronic kidney disease	498 (12.4%)	OR 1.15 (0.69–1.91); *p* = 0.588
Chronic hemodialysis	76 (1.9%)	OR 0.81 (0.19–3.34); *p* = 0.772
GERD/Ulcer disease	566 (14.1%)	OR 2.25 (1.51–3.37); *p* < 0.001 *
Inflammatory bowel disease	46 (1.1%)	OR 2.12 (0.65–6.95); *p* = 0.211
Chronic liver disease	110 (2.7%)	OR 1.14 (0.41–3.14); *p* = 0.799
Liver cirrhosis	49 (1.2%)	OR 1.99 (0.61–6.47); *p* = 0.255
Epilepsy	112 (2.8%)	OR 1.74 (0.75–4.04); *p* = 0.198
Mental retardation	45 (1.1%)	OR 1.41 (0.34–5.87); *p* = 0.639
Schizophrenia	60 (1.5%)	OR 1.04 (0.25–4.3); *p* = 0.958
Dementia	829 (20.7%)	OR 1.02 (0.66–1.56); *p* = 0.937
Active malignant disease	429 (10.7%)	OR 1.76 (1.9–2.81); *p* = 0.019 *
Metastatic malignant disease	280 (7%)	OR 1.13 (0.58–2.17); *p* < 0.001 *
History of malignant disease	718 (17.9%)	OR 1.42 (0.93–2.15); *p* = 0.102
Thyroid disease	371 (9.2%)	OR 0.82 (0.43–1.58); *p* = 0.553
Autoimmune/rheumatic dis.	174 (4.3%)	OR 1.08 (0.47–2.48); *p* = 0.858
Asthma	119 (3%)	OR 0.77 (0.24–2.47); *p* = 0.664
COPD	286 (7.1%)	OR 1.1 (0.57–2.12); *p* = 0.779
Transplanted organ	43 (1.1%)	-
Trauma/surgery 1 month prior to or during hospitalization	526 (13.1%)	OR 2.07 (1.36–3.15); *p* < 0.001 *
Known thrombophilia	21 (0.5%)	OR 3.2 (0.74–13.9); *p* = 0.119
Anticoagulant therapy	585 (14.6%)	OR 1.81 (1.27–2.6); *p* < 0.001 *
Aspirin	765 (19.1%)	OR 1.24 (0.81–1.89); *p* = 0.315
Steroids prior to admission	489 (12.2%)	OR 1.17 (0.71–1.95); *p* = 0.523
Antipsychotics	413 (10.3%)	OR 1.06 (0.6–1.87); *p* = 0.830
Antidepressants	288 (7.2%)	OR 1.6 (0.91–2.83); *p* = 0.103
Active chemotherapy	101 (2.5%)	OR 0.92 (0.29–2.94); *p* = 0.883
Statin	962 (24%)	OR 1.28 (0.87–1.89); *p* = 0.203
Hormonal therapy	92 (2.3%)	OR 1.02 (0.41–2.54); *p* = 0.964

* statistically significant at level *p* < 0.05. Abbreviations: HR—hazard ratio; CI—confidence interval; VTE—venous thromboembolism; GERD—gastroesophageal reflux disease; COPD—chronic obstructive pulmonary disease.

**Table 2:** Laboratory parameters on admission, and their relationship with major bleeding.

	Overall (N = 4014)	OR with 95% CI for Major Bleeding
IL-6 (pg/mL)	53.4 IQR (20.9–121.8)	OR 0.99 (0.99–1.0); *p* = 0.377
Procalcitonin (ng/mL)	21.5 IQR (0.09–0.76)	OR 1.02 (1.0–1.03); *p* = 0.014 *
WBC (x10^9^/L)	8 IQR (5.7–11.2)	OR 1.03 (1.01–1.04); *p* < 0.001 *
Hemoglobin (g/L)	128 IQR (113–141)	OR 0.96 (0.96–0.98); *p* < 0.001 *
MCV (fL)	88.9 IQR (85.6–92.2)	OR 1.0 (0.98–1.04); *p* = 0.608
MCHC (g/L)	333 IQR (324–340)	OR 0.98 (0.98–1.0); *p* = 0.161
RDW (%)	14.1 IQR (13.4–15.2)	OR 1.16 (1.09–1.23); *p* < 0.001 *
Platelets (x10^9^/L)	220 IQR (163–296)	OR 0.99 (0.99–1.0); *p* = 0.123
CRP (mg/L)	88.2 IQR (39.5–150.8)	OR 0.99 (0.99–1.0); *p* = 0.127
Ferritin (µg/L)	711 IQR (386–1290)	OR 1.0 (0.99–1.0); *p* = 0.377
D-dimers (mg/L FEU)	1.42 IQR (0.73–3.58)	OR 1.12 (0.96–1.29); *p* = 0.134
eGFR (ml/min/1.73 m^2^)	71.6 IQR (45.8–90.4)	OR 0.98 (0.98–0.99); *p* < 0.001 *
LDH (U/L)	335 IQR (248–453)	OR 1.0 (0.99–1.0); *p* = 0.241
AST (U/L)	41 IQR (28–64)	OR 1.0 (0.99–1.0); *p* = 0.972
ALT (U/L)	31 IQR (19–52)	OR 1.0 (0.99–1.0); *p* = 0.916
GGT (U/L)	42 IQR (24–81)	OR 0.99 (0.99–1.0); *p* = 0.523
ALP (U/L)	72 IQR (56–97)	OR 1.0 (0.99–1.0); *p* = 0.695
Total bilirubin (µmol/L)	11.4 IQR (8.6–15.9)	OR 1.0 (1.0–1.0); *p* = 0.0519
Albumin (g/L)	32 IQR (28–35)	OR 0.89 (0.86–0.94); *p* < 0.001 *
PT (%)	100 IQR (89–109)	OR 0.98 (0.97–0.99); *p* = 0.016 *

* statistically significant at level *p* < 0.05.

**Table 3:** COVID-19 disease severity- and hospitalization-related parameters, and their relationship with major bleeding.

	Overall (N = 4014)	OR with 95% CI for Major Bleeding
Origin of referral		
Home	1477 (36.8%)	Reference category
Nursing home	493 (12.3%)	OR 1.2 (0.6–2.37); *p* = 0.592
Other hospital	2044 (50.9%)	OR 2.14 (1.41–3.27); *p* < 0.001 *
Day of disease on admission	5 IQR (1–9)	OR 0.6 (0.94–1.0); *p* = 0.065
ECOG status on admission	3 IQR (1–4)	OR 1.3 (1.12–1.51); *p* < 0.001 *
Pneumonia	3531 (88%)	OR 0.96 (0.57–1.6); *p* = 0.896
Bilateral pneumonia	2600 (64.8%)	OR 0.79 (0.56–1.14); *p* = 0.220
Oxygen therapy	3265 (81.3%)	OR 1.11 (0.7–1.78); *p* = 0.634
MEWS score	2 IQR (1–4)	OR 1.08 (0.98–1.17); *p* = 0.092
Symptom severity		
Mild	449 (11.2%)	Reference category
Moderate	206 (5.1%)	OR 0.53 (0.18–1.62); *p* = 0.269
Severe	2761 (68.8%)	OR 0.77 (0.45–1.34); *p* = 0.365
Critical	598 (14.9%)	OR 1.53 (0.83–2.82); *p* = 0.174
Other infection on admission	587 (14.6%)	OR 1.65 (1.08–2.53); *p* = 0.022 *
Length of hospitalization (days)	10 IQR (6–16)	OR 1.04 (1.03–1.05); *p* < 0.001 *
Intensive care unit	913 (22.7%)	OR 4.01 (2.82–5.7); *p* < 0.001 *
High-flow oxygen th.	771 (19.2%)	OR 1.86 (1.27–2.74); *p* = 0.002 *
Mechanical ventilation	675 (16.8%)	OR 3.18 (2.21–4.59); *p* < 0.001 *
Immobilization ≥7 days	1769 (44.1%)	OR 2.52 (1.74–3.65); *p* < 0.001 *
Venous thromboembolism	215 (5.3%)	OR 1.35 (0.67–2.69); *p* = 0.399
Pulmonary embolism	145 (3.6%)	OR 1.08 (0.43–2.68); *p* = 0.871
Deep venous thrombosis	86 (2.1%)	OR 1.48 (0.54–4.11); *p* = 0.448
Arterial thrombosis	233 (5.8%)	OR 2.03 (1.15–3.61); *p* = 0.046 *
Acute myocardial infarction	68 (1.7%)	OR 0.45 (0.06–3.23); *p* = 0.424
Acute cerebrovascular insult	111 (2.8%)	OR 1.76 (0.76–4.08); *p* = 0.190
LMWH therapy	3447 (85.9%)	OR 0.89 (0.54–1.45); *p* = 0.647
Intensified LMWH therapy	1369 (34.1%)	OR 1.9 (1.19–3.02); *p* = 0.007 *
Corticosteroid therapy	2792 (69.6%)	OR 1.01 (0.69–1.48); *p* = 0.958
Intensified corticosteroid th.	1157 (28.8%)	OR 1.43 (0.99–2.06); *p* = 0.054

* statistically significant at level *p* < 0.05.

## Data Availability

Data are available per reasonable request.
